# Electrolyte Imbalance Among Bangladeshi Patients With COVID-19

**DOI:** 10.7759/cureus.35352

**Published:** 2023-02-23

**Authors:** Shafia Sharmin Moutushi, Taslima Akter, Md. Ahsanul Haq, Rahnuma Ahmad, Susmita Sinha, Nihad Adnan, Mainul Haque

**Affiliations:** 1 Biochemistry, Holy Family Red Crescent Medical College, Dhaka, BGD; 2 Microbiology, Holy Family Red Crescent Medical College, Dhaka, BGD; 3 Biostatistics, Infectious Diseases Division, International Centre for Diarrhoeal Disease Research, Bangladesh (icddr,b), Dhaka, BGD; 4 Physiology, Medical College for Women and Hospital, Dhaka, BGD; 5 Physiology, Khulna City Medical College and Hospital, Khulna, BGD; 6 Microbiology, Jahangirnagar University, Savar Union, BGD; 7 Pharmacology and Therapeutics, National Defence University of Malaysia, Kuala Lumpur, MYS

**Keywords:** retrospective study, imbalance, mortality, morbidity, bangladesh, low blood k+, low blood na+, coronavirus disease 2019, electrolytes, serum

## Abstract

Introduction

Infection with SARS-CoV-2 begins in the lower respiratory tract, but COVID-19 often involves the renal system, resulting in serum electrolyte imbalance. Monitoring serum electrolyte levels and parameters of liver and kidney function is essential to understand disease prognosis.

Objectives

This study aimed to determine the effect of imbalances in serum electrolytes and other parameters on COVID-19 severity.

Material and method

This retrospective study comprised 241 patients, ages 14 years and older, including 186 patients who were moderately affected and 55 who were categorized as severely affected by COVID-19. Serum electrolytes (sodium (Na^+^), potassium (K^+^), and chloride (Cl^−^)) and biomarkers of kidney and liver function (creatinine and alanine aminotransferase (ALT)) were measured and correlated with disease severity. This research was conducted among admitted patients of Holy Family Red Crescent Medical College Hospital designated into two groups based on retrospective hospital records. Individuals with moderate illness had evidence of lower respiratory tract infection (cough, cold, breathless, etc.) during clinical assessment or imaging (chest X-ray and computed tomography (CT) scan of the lungs) and have an oxygen saturation by pulse oximetry (SpO_2_) ≥ 94% on room air at sea level. The severely ill group involved individuals with SpO_2_ ≤94% on room air at sea level and respiratory rate ≥ 30 breaths/minute, and critically ill patients are those who needed mechanical ventilation or required intensive care unit (ICU) care. This categorization was based on the Coronavirus Disease 2019 (COVID-19) Treatment Guidelines (https://www.covid19treatmentguidelines.nih.gov/about-the-guidelines/whats-new/).

Results

Average Na^+^ and creatinine increased by 2.30 parts (95% confidence interval (CI) = 0.20, 4.81, P = 0.041) and 0.35 units (95% CI = 0.03, 0.68, P = 0.043) in severe cases compared with moderate cases. Older participants had relatively Na^+^ lowered to -0.06 parts (95% CI = -0.12, -0.001, P = 0.045), significant Cl^−^ reduction by 0.09 units (95% CI = -0.14, -0.04, P = 0.001), and ALT by 0.47 units (95% CI = -0.88, -0.06, P = 0.024), whereas serum creatinine was increased by 0.01 parts (95% CI = 0.001, 0.02, P = 0.024). The creatinine and ALT of COVID-19 participants were significantly higher in males by 0.34 units and 23.2 units, respectively, compared with females. In severe COVID-19 cases compared with moderate cases, the risks of hypernatremia, elevated chloride levels, and elevated serum creatinine levels were increased by 2.83-fold (95% CI = 1.26, 6.36, P = 0.012), 5.37-fold (95% CI = 1.90, 15.3, P = 0.002), and 2.00-fold (95% CI = 1.08, 4.31, P = 0.039), respectively.

Conclusion

Serum electrolyte and biomarker levels can serve as good indicators of the condition and disease prognosis of patients with COVID-19. Our purpose in this study was to determine the association between serum electrolyte imbalance and disease severity. We collected data from ex post facto hospital records and did not intend to assess the mortality rate. Consequently, this study expects that the prompt diagnosis of electrolyte disparity or disturbance possibly minimizes COVID-19-related morbidity and mortality.

## Introduction

SARS-CoV-2 causes respiratory tract infection and has led to a pandemic that has increased illness and death worldwide. The World Health Organization (WHO) named the related illness COVID-19 [1] on January 30, 2020, and proclaimed the growing epidemic a community health crisis of global concern [2]. The most common features of COVID-19 include dry cough, fever, fatigue, and muscle pain, with less common features being headache, shortness of breath, diarrhea, and abdominal discomfort [3-5].

COVID-19 has been shown to affect the cardiovascular, gastrointestinal (GI), nervous, renal, and respiratory systems. Among these systems, the GI tract and the renal system play pivotal roles in maintaining the equilibrium of body fluids and electrolytes. Disruption of these systems can lead to fluid and electrolyte imbalances that can be life-threatening if untreated [6,7], and changes in serum sodium (Na^+^), potassium (K^+^), chloride (Cl^-^), and calcium ions (Ca2^+^) have been shown to be associated with disease severity [6,7].

The renal system has extensive functions, including urine formation, hormone synthesis, blood pressure control, pH maintenance, and plasma osmolarity regulation [8]. Infection with coronaviruses is propagated via respiratory aerosol and close association or touch [9]. The viruses penetrate human cells by adhering to angiotensin-converting enzyme 2 (ACE2) receptors [10,11]. High levels of these receptors in renal cells make them especially susceptible to coronavirus invasion, and recent studies show that renal involvement is frequent in COVID-19-affected patients [3,11]. Acute kidney injury can occur among these patients because of the cytopathic end results of COVID-19 on podocytes and proximal tubular cells, leading to increased mortality risk [11]. Typically, podocytes and proximal tubular cells maintain normal kidney functions such as filtration, absorption, and secretion of substances. Notably, podocytes are more susceptible to bacterial and viral invasions [11]. Therefore, assessment of kidney function and electrolyte abnormalities is important among patients with COVID-19.

The GI system is also affected by COVID-19, with 11.4%-50% of COVID-19 patients presenting with GI symptoms [12]. Although COVID-19 mainly affects the respiratory system, the potential exists for damage to the intestinal mucosa and microbiota, thereby causing diverse GI symptoms, most commonly diarrhea, loss of appetite, abdominal discomfort, and vomiting. These symptoms hamper normal GI function and can cause electrolyte disturbance [13].

Several processes are involved in maintaining fluid and electrolyte equilibrium via the kidney and the GI pathway. Correspondingly, any interference with these systems disrupts fluid and electrolyte equilibrium [12]. COVID-19 patients often experience an extended hospital stay and usually receive multiple types of treatment, ranging from ventilatory support to experimental agents [13]. In addition, fever, sweating, hyperventilation, side effects of medicines, and changes in diet may lead to electrolyte imbalance [13]. The typical electrolyte disturbances identified in association with COVID-19 are hyponatremia, hypokalemia, and hypochloremia [12]. The most common is hyponatremia, which occurs primarily due to the syndrome of inappropriate antidiuresis (SIAD) [14]. COVID-19 infection promotes interleukin 6 (IL-6) and cytokine release, causing electrolyte disequilibrium by inducing the nonosmotic release of vasopressin [14]. Hypernatremia may increase the possibility of infection severity [15], and both hyponatremia and hypernatremia can heighten the risk of mortality among hospitalized patients [16]. Several reports on hyponatremia among COVID-19 patients at the time of admission have suggested that the low level of plasma Na^+^ at this time often predicts disease severity and mortality [14,17,18].

Apart from Na^+^, a low K^+^ level at the time of admission has been related to a poor prognosis and disease severity [19]. Previous research on COVID-19 patients indicated that hypokalemia is a frequent finding in these patients, and it may increase the risk of developing fatal arrhythmias [20,21]. Hypokalemia in SARS-CoV-2 infection may occur due to hyperactivation of the renin-angiotensin-aldosterone system, GI loss, lack of appetite because of concurrent illness, and damage of the tubules due to ischemia or nephrotoxic agents [22]. Moreover, hypokalemia can aggravate acute respiratory distress syndrome and affect myocardial function [23].

Objectives of the study

This study intended to investigate blood biomarkers and electrolyte abnormalities among Bangladeshi COVID-19 cases and determine whether a difference exists between moderately and severely affected patients admitted to Holy Family Red Crescent Medical College Hospital, Dhaka, Bangladesh, in 2021-2022. Although alteration in the biochemical parameters and electrolyte imbalance in COVID-19 infection is a fact, the type of electrolyte abnormality was not the same around the globe. Thereby, our objective was to identify the type of electrolyte imbalance in the Bangladeshi population.

## Materials and methods

Study design

This study utilized the data of admitted patients from hospital records from July 2021 to January 2022. Therefore, this was a retrospective study based on hospital archive records.

Study area and population

This study was based on COVID-19 patients hospitalized in Holy Family Red Crescent Medical College Hospital, Dhaka, Bangladesh. This hospital is one of the tertiary care hospitals in the country’s capital city and was designated as a “COVID-dedicated” hospital by the government of the People’s Republic of Bangladesh. The data utilized in the current study were from hospital records.

Study period

This research was conducted from July 2021 to January 2022.

Research subjects

Inclusion Criteria

The majority of patients were adults (in Bangladesh, 18 years and above are considered adults), and only two patients were below 18 years. COVID-19 was diagnosed according to the guideline formulated by the Ministry of Health, Dhaka, Bangladesh [24]. All patients were selected according to their symptoms, such as cough and pyrexia. SARS-CoV-2 was confirmed using the gold standard real-time reverse transcriptase-polymerase chain reaction (RT-PCR) on respiratory tract specimens [25]. RT-PCR was conducted to confirm the diagnosis before admitting the strongly suspected case to our hospital. The specimens for conducting RT-PCR tests were obtained from both the oropharynx and nasopharynx. Patients were divided into two groups based on the severity of their symptoms. The moderate group had signs and symptoms of respiratory tract infection of COVID-19 as per described in the national guideline [24], while the severe group exhibited respiratory distress (≥30 breaths/minute) and reduced oxygen saturation (≤90% at rest). The severe group included critically ill patients who needed mechanical ventilation or required admission to the intensive care unit (ICU).

Exclusion Criteria

Among 250 patients with COVID-19 who were hospitalized, nine patients with renal failure were excluded from this study. The remaining 241 patients, aged 14-95 years, were included. This study excluded only established cases of renal failure and included all other cases with major comorbidities such as diabetes mellitus, hypertension, and arthritis. Of these 241 patients, 186 were moderately affected and 55 were severely affected and needed mechanical ventilation or ICU support. Serum electrolytes were assessed using a Dimension EXL 200 autoanalyzer (Siemens, Tokyo, Japan). Patients were grouped as hyponatremic (<136 mmol/L), normonatremic (136-144 mmol/L), or hypernatremic (>144 mmol/L) based on the reference level of serum Na^+^ (136-144 mmol/L). They were additionally identified as hypokalemic (<3.7 mmol/L), normokalemic (3.7-5.1 mmol/L), or hyperkalemic (>5.1 mmol/L) based on the normal serum K^+^ level of 3.5-5.1 mmol/L [26].

Statistical analysis

Statistical analysis was carried out using STATA version 15 (StataCorp, College Station, TX, USA), and a graphical display was made using GraphPad Prism 8.3.2 (GraphPad Software, San Diego, California USA). The distribution of electrolyte, creatinine, and alanine aminotransferase (ALT) levels in moderate and severe COVID-19 cases are presented as numbers and percentages. Multiple regression models were used to estimate the effects of electrolyte, creatinine, ALT, and demographic characteristics on disease severity. Additionally, the risk of disease severity was estimated using a logistic regression model. Statistical values were reported with 95% confidence intervals (CIs), and statistical significance was considered at P < 0.05.

Ethical approval

This study acquired ethical approval from the Institutional Review Board (IRB) of Holy Family Red Crescent Medical College, Dhaka, Bangladesh, with reference number IERC/38/Res/May/2022/03/hf, dated May 3, 2022. Our study was conducted on retrospective archive hospital records. After that, there was no necessity for written informed consent from individual patients.

## Results

As we divided the patients into two groups, the mean ± standard deviation (SD) age of the moderately affected participants was 53.47 ± 18.12, and of the severe cases was 63.36 ± 18.22 (Table 1). Among the moderately affected COVID-19 patients, 47% (87) and 53% (98) were male and female, respectively. Among the severely affected group, 37.5% (21) and 62.5% (35) were male and female, respectively.

**Table 1:** Sociodemographic characteristics of the research participants Note: Data are displayed as mean ± SD or number (percentage). SD: standard deviation

Traits	Moderate cases (n = 186)	Severe cases (n = 55)
Age (years), mean ± SD	53.47 ± 18.12	63.36 ± 18.22
Age group (years), number (%)		
0-19	8 (4.30)	0 (0)
20-39	32 (17.20)	8 (14.54)
40-59	69 (37.09)	9 (16.36)
60-79	62 (33.33)	28 (50.91)
≥80	15 (8.06)	10 (18.18)
Sex, number (%)		
Male	87 (47)	21 (37.5)
Female	98 (53)	35 (62.5)

Table 2 shows the changes in electrolytes and other biomarkers in moderately and severely affected COVID-19 patients. Among the moderately affected patients, 38 (21%) had normal Na^+^ levels, 63.5% (115) had low Na^+^ levels, and 15.5% (28) had serum Na+ levels above the normal range. Among the 55 severely affected cases, 27.8% (15) had normal serum Na^+^ levels, 44.40% (24) had hyponatremia, and 27.8% (15) had hypernatremia. Regarding serum K^+^ levels, among the 186 moderately affected cases, only 24.9% (45) of the patients had normal levels, 71.8% (130) had hypokalemia, and 3.31% (6) had hyperkalemia. In comparison, 20.4% (11) of the severely affected patients had normal serum K^+^ levels, 75.9% (41) had hypokalemia, and 3.7% (2) had hyperkalemia, indicating that hypokalemia is a more typical electrolyte disturbance in COVID-19 patients. Among the other electrolytes, the CI^- ^level was below normal in both groups, with 77.9% (141) and 59.3% (32) patients having low serum CI^-^ levels in the moderately and severely affected groups, respectively. Among other biomarkers, serum creatinine was above normal in 35.1% (53) of moderately affected cases and 57.1% (24) of severely affected cases. The moderate group possessed a total of 148 cases. However, among moderate cases, serum creatinine was normal in 64.9% (96) cases. On the other hand, among the severe cases, 42% (18) of patients had normal serum creatinine levels. Subsequently, serum creatinine reflects a possible association with disease fatality. 

**Table 2:** Distribution of electrolyte, creatinine, and alanine transaminase levels in moderate and severe cases of COVID-19 COVID-19: coronavirus disease 2019

Electrolyte/biomarker	Range	Moderate, number (%)	Severe, number (%)
Sodium	Normal	38 (21)	15 (27.8)
	Hyponatremia	115 (63.5)	24 (44.4)
	Hypernatremia	28 (15.5)	15 (27.8)
Potassium	Normal	45 (24.9)	11 (20.4)
	Hypokalemia	130 (71.8)	41 (75.9)
	Hyperkalemia	6 (3.31)	2 (3.70)
Chloride	Normal	31 (17.1)	12 (22.2)
	Below	141 (77.9)	32 (59.3)
	Above	9 (4.97)	10 (18.5)
Serum creatinine	Normal	96 (64.9)	18 (42.9)
	Above	52 (35.1)	24 (57.1)
Alanine transaminase	Normal	62 (53)	14 (56)
	Above	55 (47)	11 (44)

The average Na^+^ and creatinine increased by 2.30 units (95% CI = 0.20, 4.81, P = 0.041) and 0.35 units (95% CI = 0.03, 0.68, P = 0.043) in severe cases compared to moderate cases. Older participants had relatively lower Na^+^ to -0.06 units (95% CI = -0.12, -0.001, P = 0.045), whereas a higher creatinine to 0.01 units (95% CI = 0.001, 0.02, P = 0.024). A significant reduction in Cl^-^ by 0.09 units (95% CI = -0.14, -0.04, P = 0.001) and ALT by 0.47 units (95% CI = -0.88, -0.06, P = 0.024) was also observed among older patients. The creatinine and ALT levels of COVID-19 participants were significantly higher in males by 0.34 units and 23.2 units, respectively, compared with females (Table 3).

**Table 3:** Effects of disease severity on electrolyte, creatinine, and alanine transaminase levels Note: A multiple regression model was utilized to evaluate the P-value. CI: confidence interval, COVID-19: coronavirus disease 2019

	Sodium		Potassium		Chloride		Creatinine		Alanine transaminase	
	β (95% CI)	P	β (95% CI)	P	β (95% CI)	P	β (95% CI)	P	β (95% CI)	P
COVID-19 severity										
Moderate	Reference		Reference		Reference		Reference		Reference	
Severe	2.30 (0.20, 4.81)	0.041	0.10 (-0.12, 0.32)	0.358	1.84 (-0.42, 4.11)	0.110	0.35 (0.03, 0.68)	0.043	2.82 (-16.7, 22.4)	0.775
Age	-0.06 (-0.12, -0.001)	0.045	0.001 (-0.004, 0.01)	0.567	-0.09 (-0.14, -0.04)	0.001	0.01 (0.001, 0.02)	0.024	-0.47 (-0.88, -0.06)	0.024
Sex										
Female	Reference		Reference		Reference		Reference		Reference	
Male	-1.58 (-3.62, 0.48)	0.131	0.14 (-0.03, 0.32)	0.110	-0.50 (-2.36, 1.35)	0.595	0.34 (0.05, 0.62)	0.021	23.2 (8.77, 37.7)	0.002

**Figure 1:**
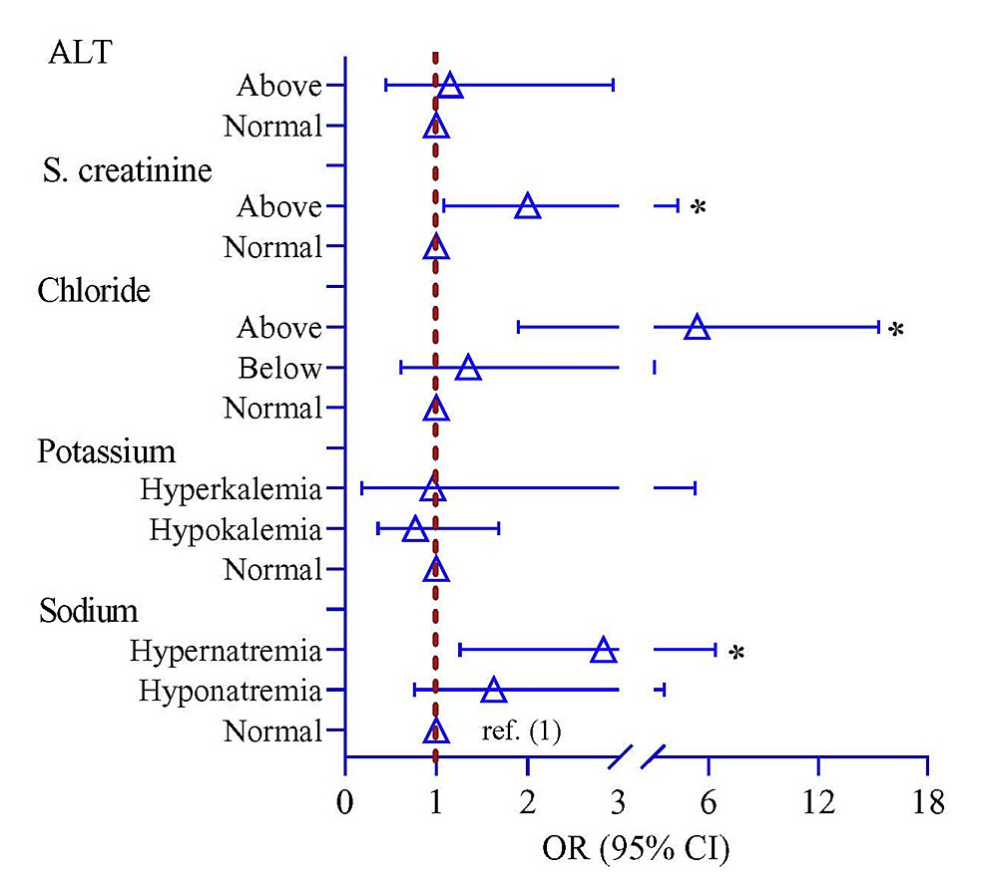
Risk of disease severity in moderate compared with severe COVID-19 cases regarding electrolyte, creatinine, and alanine transaminase levels Note: A logistic regression model was utilized to evaluate the p-value. The regression model was adjusted by age and sex. ALT: alanine transaminase, S: serum, ref.: reference, OR: odds ratio, CI: confidence interval Figure credit: Md. Ahsanul Haq

The risk of hypernatremia, higher Cl^-^, and serum creatinine above normal was higher by 2.83-fold (95% CI = 1.26, 6.36, P = 0.012), 5.37-fold (95% CI = 1.90, 15.3, P = 0.002), and 2.00-fold (95% CI = 1.08, 4.31, P = 0.039), respectively, in severe COVID-19 cases compared with moderate cases (Table 4, Figure 1).

**Table 4:** Risk of disease severity in moderate compared with severe participants on electrolyte, creatinine, and alanine transaminase levels Note: A logistic regression model was used to estimate the P-value. The regression model was adjusted by age and sex. OR: odds ratio, CI: confidence interval

Electrolyte/biomarker	Range	OR (95% CI)	P-value
Sodium	Normal	1	
	Hyponatremia	1.63 (0.76, 3.56)	0.210
	Hypernatremia	2.83 (1.26, 6.36)	0.012
Potassium	Normal	1	
	Hypokalemia	0.77 (0.36, 1.68)	0.514
	Hyperkalemia	0.96 (0.18, 5.26)	0.962
Chloride	Normal	1	
	Below	1.35 (0.61, 3.03)	0.464
	Above	5.37 (1.90, 15.3)	0.002
Serum creatinine	Normal	1	
	Above	2 (1.08, 4.31)	0.039
Alanine transaminase	Normal	1	
	Above	1.15 (0.45, 2.94)	0.773

## Discussion

Multiple studies regarding COVID-19 including meta-analysis revealed that older age, especially 60 years or more, as well as associated diabetes mellitus and hypertension, had higher disease severity than ages below 60 years [27-30]. Sex also plays a role among patients suffering from COVID-19 with poor prognosis [31]. Several studies reported that patients below 41.3-56.9 years old suffer from not as many fatal issues as patients aged 46.1-67.1 years [30,32]. Consequently, COVID-19 disease fatality frequently depends on the aging process, sex, multiple comorbidities, and the need for polypharmacy because of several diseases in the elderly community [33]. Our study similarly observed that with regard to the demographic profiles of the patients, severity increased with age, as mentioned in earlier published papers.

**Figure 2:**
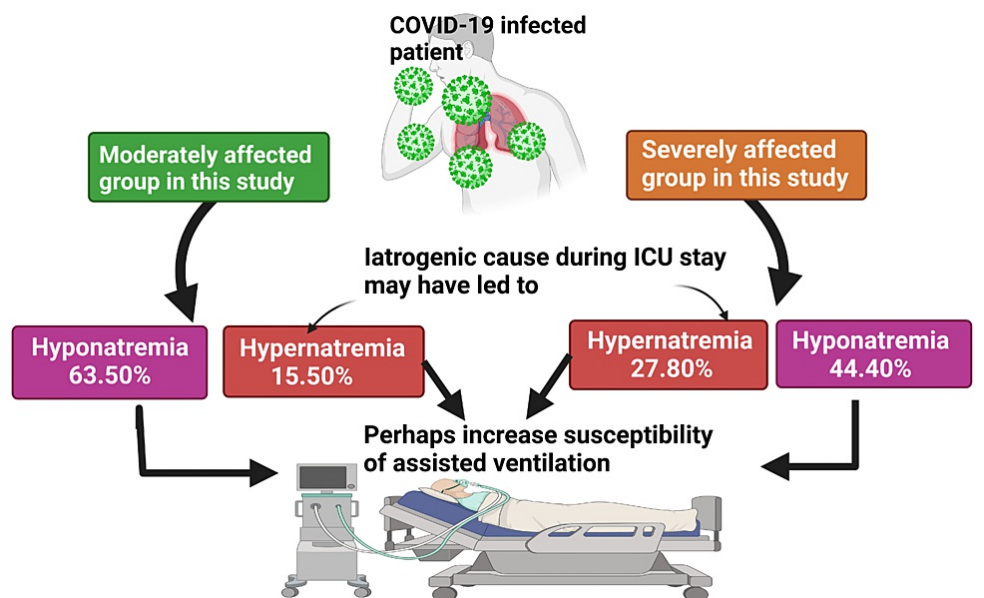
Findings regarding the sodium level in blood in moderately and severely affected COVID-19 patients Note: Hypernatremia is not a complication of COVID-19, but rather, it may be due to iatrogenic causes taking place during their stay in the ICU. Also, hyponatremia and hypernatremia may lead to increased susceptibility to assisted ventilation. ICU: intensive care unit, COVID-19: coronavirus disease 2019 This figure was developed using the premium version of BioRender (https://biorender.com/) with license number ﻿RS24WAZAK4. Image credit: Rahnuma Ahmad

In this study, we found that COVID-19 is associated with both hyponatremia (below 135 mEq/L) and hypernatremia (below 145 mEq/L). Among the patients in the moderately affected group, 63.50% of cases had hyponatremia and 15.50% had hypernatremia. In the group of patients with severe COVID-19, 44.40% had hyponatremia and 27.8% experienced hypernatremia. In another study, 57% of patients with severe COVID-19 had hyponatremia on admission and 2% had hypernatremia, and 42% developed hypernatremia two weeks after hospitalization [21]; these percentages are higher than those in our study. In several other studies, 25%-52% of COVID-19 patients had hyponatremia on admission [13,16,17]. In these studies, decreased levels of serum Na^+^ were associated with an increased need for assisted ventilation but were unrelated to death [13,16,17]. Although hyponatremia was common at the time of admission to the ICU, the Na^+^ level gradually increased as patients received Na^+^-containing fluid, and hyponatremia was rare at the time of death [21]. Given that hyponatremia was common at admission, with hypernatremia developing later, a high Na^+^ level was less likely to be caused by the disease [21]. A few other studies have found a prevalence of hypernatremia of 4.3%-15.8% among ICU patients, with Na^+^ levels of 145-150 mmol/L [23,34]. The cause of ICU-related hypernatremia is often iatrogenic [35] and increases hospital stay [36], the requirement for assisted ventilation [37,38], and the risk of mortality (Figure 2) [37,38]. Therefore, the Na^+^ level was proposed as an indicator of disease severity.

The current study has another critical finding: hypokalemia was prevalent among COVID-19 patients, affecting 71.8% of moderate and 75.9% of severe cases. Although there was hypokalemia in both groups, there was no statistically significant difference (P > 0.05). Therefore, we can interpret that irrespective of disease severity, serum K^+^ level decreases in both cases. Another study mentioned hypokalemia as an impending expression of COVID-19 [19] as a consequence of the interaction of SARS-CoV-2 with the renin-angiotensin-aldosterone system [18]. One more research study revealed that hypokalemia was detected in 41% (119) of cases out of 290 patients on admission. Their average K^+^ concentration was 3.1 ± 0.1 mmol/L [39]. In our study, the mean serum K^+^ concentration in moderate cases was 3.14 ± 0.33 mmol/L, while in severe cases, it was 2.97 ± 0.53 mmol/L. This finding indicates that a low level of K^+^ is associated with disease severity; however, the difference was not statistically significant.

**Figure 3:**
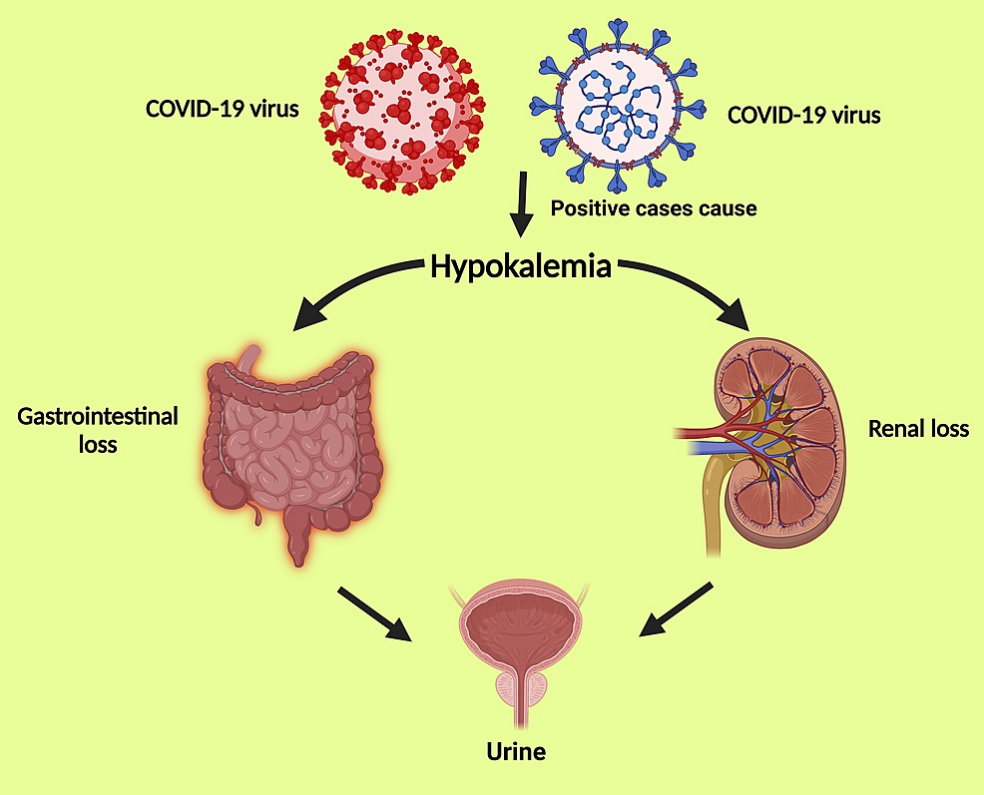
Effects of hypokalemia in COVID-19-positive subjects COVID-19: coronavirus disease 2019 This figure was developed using the premium version of BioRender (https://biorender.com/) with license number SM24WDI4FM. Image credit: Susmita Sinha

A previous study [3] that included 41 patients reported that the mean serum K^+^ was 4.6 mmol/L in severe cases and 4.1 mmol/L in moderate cases, indicating that an increase in serum K^+^ was related to the severity of the disease. Our outcomes, which were based on a larger number of patients, did not accord with those findings. In another study, hypokalemia was prevalent in 54% (95 of 175) of COVID-19-positive cases, with serum K^+^ concentration being less than 3.5 mmol/L [18]. Another study asserted that hypokalemia was a day-to-day finding among 62% of COVID-19 cases, affecting 108 out of 175 patients. These patients had a mean serum K^+ ^of below 3.5 mmol/L, and only 10% of patients had K^+ ^levels above 4 mmol/L [18]. Increased GI and renal losses of K^+ ^are probably the cause of hypokalemia in COVID-19 [39,40]. As diarrhea is a less common feature [1,41], urinary loss is possibly the primary cause of hypokalemia in our study. However, urinary K^+^ excretion in this study was not conducted due to structural and financial constraints. Subsequently, this research was unable to ascertain the cause of hypokalemia. As hypokalemia potentially stresses cardiac function, prompt therapeutic intervention is absolutely required to accomplish a better clinical end result (Figure 3).

Among the other electrolytes, the CI^- ^level was below average in both groups. Of the cases, 77.9% (141) and 59.3% (32) had lower serum CI^-^ levels in moderately and severely affected patients, respectively. Among the other biomarkers, serum creatinine was higher than average in 35.1% (52) and 57.1% (24) of the moderately and severely affected cases, respectively, indicating that the serum creatinine level reflects disease severity. The risk of hypernatremia, hyperchloremia, and higher creatinine is higher in severe cases when compared with moderate cases. Therefore, multiple studies reported that assessment of serum electrolytes remains a good diagnostic marker to determine patient disease severity and prognosis, especially among COVID-19 cases [42-44].

Limitations of the study

Our research has a certain amount of shortcomings, as several risk factors are related to the severity of COVID-19. Therefore, confounding factors may have affected the results. Nevertheless, as this was based on retrospective hospital records, the researchers did not have the opportunity to detect the association between confounding variables. We took only on-admission levels of electrolytes. The principal investigator does not have any access to data on fluid intake and output before admission. Moreover, because the study was based on retrospective hospital records, it did not have the prospect of following up on the test results. Consequently, we cannot report the patients’ outcomes.

## Conclusions

Electrolyte balance is crucial for sustaining health and maintaining normal physiology. Even a slight divergence from average electrolyte concentrations may worsen a patient’s condition and increase the possibility of death. In COVID-19, various forms of kidney and GI tract involvement are common, and any interference with these systems can lead to fluid and electrolyte disturbance. We have found hyponatremia, hypernatremia, hypokalemia, and hypochloremia as the most common electrolyte disorders in COVID-19. Serum electrolyte concentration evaluation remains a good appraisal tool to assess the patient’s condition and disease prognosis as mentioned in earlier studies. We recommend that if electrolyte imbalances are observed, especially in outpatient settings, patients must be straightaway hospitalized and definitive therapeutic intervention instantly installed.
